# NK Cells in the Tumor Microenvironment as New Potential Players Mediating Chemotherapy Effects in Metastatic Melanoma

**DOI:** 10.3389/fonc.2021.754541

**Published:** 2021-10-12

**Authors:** Cinzia Garofalo, Carmela De Marco, Costanza Maria Cristiani

**Affiliations:** Department of Experimental and Clinical Medicine, “Magna Græcia” University of Catanzaro, Catanzaro, Italy

**Keywords:** NK cells, melanoma, chemotherapy, tumor microenvironment (TME), nanocarriers

## Abstract

Until the last decade, chemotherapy was the standard treatment for metastatic cutaneous melanoma, even with poor results. The introduction of immune checkpoints inhibitors (ICIs) radically changed the outcome, increasing 5-year survival from 5% to 60%. However, there is still a large portion of unresponsive patients that would need further therapies. NK cells are skin-resident innate cytotoxic lymphocytes that recognize and kill virus-infected as well as cancer cells thanks to a balance between inhibitory and activating signals delivered by surface molecules expressed by the target. Since NK cells are equipped with cytotoxic machinery but lack of antigen restriction and needing to be primed, they are nowadays gaining attention as an alternative to T cells to be exploited in immunotherapy. However, their usage suffers of the same limitations reported for T cells, that is the loss of immunogenicity by target cells and the difficulty to penetrate and be activated in the suppressive tumor microenvironment (TME). Several evidence showed that chemotherapy used in metastatic melanoma therapy possess immunomodulatory properties that may restore NK cells functions within TME. Here, we will discuss the capability of such chemotherapeutics to: i) up-regulate melanoma cells susceptibility to NK cell-mediated killing, ii) promote NK cells infiltration within TME, iii) target other immune cell subsets that affect NK cells activities. Alongside traditional systemic melanoma chemotherapy, a new pharmacological strategy based on nanocarriers loaded with chemotherapeutics is developing. The use of nanotechnologies represents a very promising approach to improve drug tolerability and effectiveness thanks to the targeted delivery of the therapeutic molecules. Here, we will also discuss the recent developments in using nanocarriers to deliver anti-cancer drugs within the melanoma microenvironment in order to improve chemotherapeutics effects. Overall, we highlight the possibility to use standard chemotherapeutics, possibly delivered by nanosystems, to enhance NK cells anti-tumor cytotoxicity. Combined with immunotherapies targeting NK cells, this may represent a valuable alternative approach to treat those patients that do not respond to current ICIs.

## 1 Introduction

Cutaneous melanoma is considered as the deadliest form of skin cancer because of its high tendency to metastatic spread. While *in situ* lesions can be effectively cured by surgical removal, the treatment of the metastatic disease is still challenging. Until the last decade, the standard therapy for metastatic melanoma was chemotherapy, based on the usage of three classes of drugs: alkylating agents, mitotic inhibitors and alkylating-like drugs (1).

The alkylating agents dacarbazine (DTIC) and temozolomide (TMZ) were the most widely used and effective chemotherapeutic agents used for the treatment of metastatic melanoma, with a general good tolerance and limited side effects which include modest bone marrow suppression (1). However, the outcome was poor, with ORRs around 20%, response duration of 4-6 months and complete responses observed only in 5% of treated patients (2, 3). The mitotic inhibitors paclitaxel (PTX) and docetaxel (DTX) belong to taxanes and were both associated with ORRs and survival rates similar to those observed with DTIC. However, they caused more severe side effects, including myelosuppression, hypersensitivity reactions and peripheral neuropathy, thus they were generally used as second-line therapy (1, 2). Along with PTX, the activity of the alkylating-like agent cisplatin as a first-line single therapy showed modest results (2). Furthermore, important side effects and several mechanisms of drug resistance development were associated to cisplatin usage, thus the drug was generally used in combinatorial regimens (1).

Overall, the effects of chemotherapy in metastatic melanoma were modest, mainly palliative and poorly effective in terms of survival. The picture has been dramatically changed by the introduction of immunotherapy, particularly by the immune checkpoints inhibitors (ICIs). The first ICI approved by FDA in 2011 was Ipilimumab, a monoclonal antibody (mAb) targeting the Cytotoxic T-Lymphocyte Antigen 4 (CTLA-4), which increased ORR to 19% and 5-years survival to 20% (4). The introduction of Nivolumab and Pembrolizumab, targeting the Programmed cell death protein 1 (PD-1), further increased ORRs and 5-year survival rates to 40% and 30-40%, respectively (5–7). Even more striking results were reached combining Nivolumab and Ipilimumab, whose subsequential administration in metastatic melanoma patients gave 45% and 60% of ORRs and 5-year survival, respectively (7, 8). However, a large portion of metastatic melanoma patients still does not respond to current treatments, highlighting the need to develop further therapeutical approaches. It has been proposed that this lack of effectiveness may be due to the strong immunosuppression exerted in the tumor microenvironment (TME) by both cancer and stromal cells. Indeed, immunosuppression is nowadays considered as a hallmark of cancer (9). Thus, it is likely that strategies able to counteract such immune suppression and to generate a supportive TME would represent valuable approaches to improve ICIs effectiveness.

In this context, several lines of evidence showed that, together with the well-known cytotoxic activities, chemotherapeutic drugs also display the capability to affect immune responses against tumors. These immunomodulatory properties mainly rely on five mechanisms: 1) shrinkage of the cancer mass, which reduces the systemic immune suppression induced by the tumor, 2) enhancement of the expression and/or presentation of tumor antigens, which increases cancer cell antigenicity, 3) emission of danger signals by damaged cells, which augments cancer cell immunogenicity, 4) induction of stress signals on cell surface, which increase the susceptibility of cancer cells to be killed by immune cells, 5) direct effects on immune and stromal cells within the TME (10). In addition, even the lympho- and mielodepletion, generally regarded as an undesired collateral effect of chemotherapy, may offer a therapeutic advantage by resetting the immune system and favoring the appearance and/or expansion of immune cell subsets with anti-tumor activities. It has been proposed to occur through homeostatic proliferation, that is the peripheral expansion of specific immune cell clones induced by targeted stimuli such as cytokines and/or antigens (11, 12). Thus, the combination with chemotherapy may be a promising approach to improve the outcome of metastatic melanoma patients treated with ICIs. Indeed, recent reports indicated that the standard chemotherapeutics used in metastatic melanoma can both cooperate with ICIs, increasing response rates compared to single-agent mAbs, and sensitize patients that do not respond when ICIs are administrated as first-line therapies (13–17). However, the molecular mechanisms underlying this synergism are still not fully elucidated.

Although the main target of current immunotherapies has been T cells, increasing attention is being given to Natural Killer (NK) cells because of their alternative mechanism to recognize cancer cells. However, their usage in clinical settings is limited by the same issues observed for T cells and needs to be implemented, possibly by combinatorial approaches. Here, we discuss the immunomodulatory properties of anti-melanoma chemotherapeutic drugs that could be exploited to enhance NK cells anti-tumoral functions within melanoma TME. Furthermore, we discuss the possibility to further improve such properties by the usage of nanocarriers for the targeted release of chemotherapeutic drugs.

## 2 The Role of Natural Killer Cells in Melanoma Recognition

NK cells belong to Innate Lymphoid Cells (ILCs), a family of innate lymphocytes largely contributing to tissue homeostasis. Particularly, NK cells are considered the cytotoxic arm of ILCs and, more in general, of innate immunity, thus playing a pivotal role in mounting early defenses against stressed, viral-infected and tumor-transformed cells (18). Phenotypically, NK cells are characterized by the lack of T cell lineage marker CD3 and the expression of CD56, which further distinguishes NK cells in two main subpopulations. CD56^bright^ NK cells are poorly cytolytic and mainly immunoregulatory, while CD56^dim^ NK cells display strong cytotoxicity but low secretory capabilities (19).

Peripheral blood NK cells have been shown to be widely affected by melanoma, as indicated by the association between NK cell modifications and melanoma progression and/or response to therapy (20). In melanoma, NK cells are recruited by the inflammatory chemokines CCL5 and CXCL9-11, expressed within the TME, by the engagement of the cognate receptors CCR5 and CXCR3, respectively (21). However, infiltrating NK cells are usually poor and clusterized around the stroma, not in direct contact with tumor cells (22, 23). Indeed, NK cells have been proposed to contribute to anti-melanoma responses mainly by the elimination of tumor cells spreading throughout blood circulation, particularly cancer stem cells, thus limiting metastasization (20, 24, 25). Still, the presence of NK cells in melanoma TME has been associated with tumor regression and good prognosis (22).

**Figure 1:**
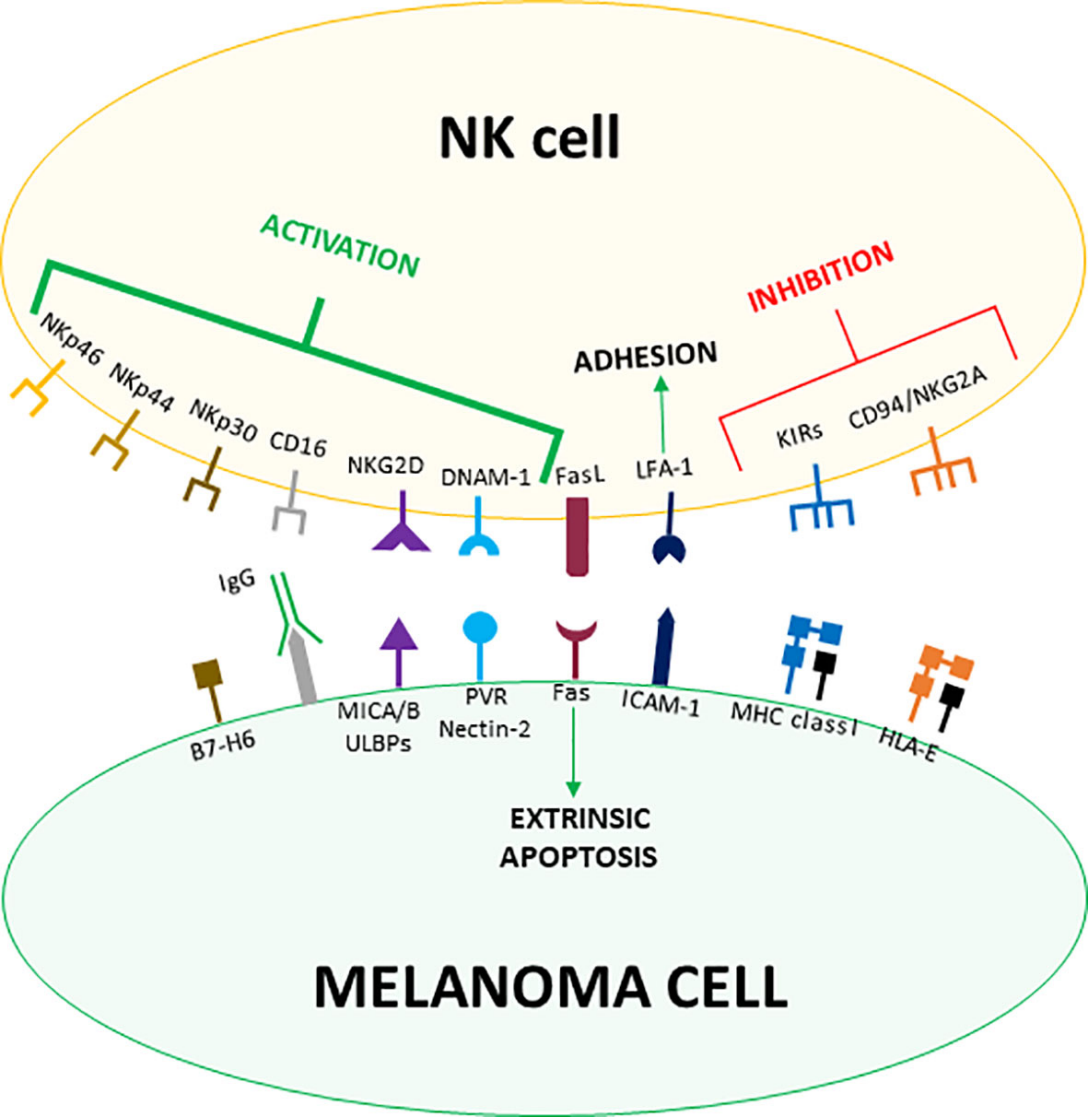
Receptor-ligand pairs involved in melanoma cells recognition by NK cells. Both MHC class I and MHC-like HLA-E molecules expressed by melanoma cells counteract NK cells activation by engaging the inhibitory receptors KIRs and CD94/NKG2A, respectively. On the other hand, malignant transformation induces the expression of NKG2D, DNAM-1 and NCR-ligands on melanoma cells, which are recognized by the cognate activating receptors expressed on NK cells. Melanoma cells usually up-regulate the expression of activating ligands and down-regulate MHC class I molecules levels tipping the balance towards the activation of NK cells. Less specific interactions such as LFA-1/ICAM-1 mediate the firm adhesion between NK and melanoma cells whereas Fas/FasL binding activates the extrinsic apoptosis. Moreover, CD16 contributes to melanoma cell killing by mediating ADCC.

As part of the innate immune system, NK cells recognize cells to eliminate through germline-encoded receptors that engage poorly polymorphic molecules/determinants expressed on target cells. Such ligands can display either inhibitory or activating effects and are indeed recognized by inhibitory or activating receptors, respectively. Thus, the activation and killing of NK cells will depend on the balance between the signals delivered by the different receptors, as stated by the “missing-self hypothesis”. Accordingly, the expression of inhibitory molecules will spare cells from killing, while inhibitory ligands down-regulation and/or activating ligands over-expression will induce NK cell cytotoxicity (26) (**Figure 1**).

The main inhibitory molecules for NK cells are classical Major Histocompatibility Complex (also known as Human Leucocyte Antigen, HLA, in humans) class I (MHC-I) molecules recognized by the Killer-cell Immunoglobulin-like Receptors (KIRs), which represent the most important inhibitory receptors expressed by NK cells. Of notice, KIRs interact with common determinants shared by HLA-A, -B, and -C molecules. While MHC-I molecules are expressed by the majority of healthy cells, they are usually down-regulated in infected or transformed cells, avoiding the engagement of KIRs and thus inducing NK cells activation (27). Indeed, down-regulation of MHC-I molecules has been widely described in melanoma and it is regarded as one of the major mechanisms determining NK cell-mediated killing of melanoma cells (28) (**Figure 1**).

Another important NK cell inhibitory receptor is CD94/Natural Killer Group (NKG) 2A, belonging to the heterodimeric C-type lectin NKG2 receptor family. CD94/NKG2A binds to HLA-E, a poorly polymorphic non-classical MHC-I molecule loaded with peptides deriving from the leader sequence of the other MHC-I molecules. Thus, CD94/NKG2A allows NK cells to sense the overall expression levels of MHC-I molecules on target cells (29) (**Figure 1**).

While inhibitory signals for NK cells are primarily mediated by MHC-I molecules, activating ligands are more heterogeneous and induced by cellular stress. The main class of NK cells activating receptors are the Natural Cytotoxicity Receptors (NCRs), consisting of three Ig-like proteins whose ligands are still poorly defined. NK cells constitutively express two NCRs, NKp30 and NKp46, while the third member of the family, NKp44, can be found on NK cell surface only after activation. Collectively, NCRs are the most important receptors triggering NK cell-mediated killing of cancer cells. Indeed, their expression correlates with the magnitude of the NK cell cytolytic activity (30) (**Figure 1**).

Another fundamental receptor involved in NK cell activation is NKG2D. While belonging to the NKG2 family, NKG2D stands out for being a monomeric receptor. Ligands recognized by NKG2D include two types of MHC-like molecules, MHC class I chain-related protein (MIC) A and B and unique long 16-binding proteins (ULBPs). Both the families of ligands are generally not found on healthy cells but are induced by stresses such as malignant transformation (31, 32). The DNAX accessory molecule-1 (DNAM-1) is a co-receptor enhancing NK cell activation triggered by the other activating receptors. DNAM-1 recognizes two ligands, CD155 (poliovirus receptor, PVR) and CD112 (Nectin-2), that are widely expressed by cancer cells (33). Moreover, NK cells can be activated through the engagement of CD16, which binds the Fc portion of IgG antibodies and thus allows NK cells to mediate the antibody-dependent cell cytotoxicity (ADCC) against opsonized target cells (34) (**Figure 1**).

The analysis of large panels of melanoma cells showed that ligands for NKG2D (MICA/B, ULBPs) and DNAM-1 (PVR, Nectin-2) are widely expressed by melanoma cells. Of them, MICA/B were more frequently observed compared to ULPBs (35, 36) and PVR was widely found, while Nectin-2 was scarcely expressed (35). The expression of NCR ligands has been also reported, though to a lesser extent (37, 38).

Either an over-expression of activating ligands or a loss of inhibitory signals triggers the main cytotoxic pathway of NK cells, that is the release of cytolytic granules, specialized secretory organelles containing perforin and granzymes inducing apoptotic cell death. Specifically, granzymes are serine proteases able to cleave and activate several intracellular proteins involved in apoptosis induction, while perforin generates pores on eucaryotic cell membrane allowing granzymes entry within the target cell (39).

Though the balance between inhibitory and activating signals represents the main factor determining target recognition and cytotoxicity, both the processes are strengthened by accessory molecules involved in cell adhesion. Among them, the β2-integrin Leucocyte Functional Antigen (LFA)-1, recognizing the Intercellular Adhesion Molecule (ICAM)-1, is found to be expressed on melanoma cells (40, 41), where it plays a central role in mediating the firm adhesion between NK and target cells as well as the polarized delivery of cytotoxic granules (42). Additionally, NK cells can eliminate targets by inducing the extrinsic apoptotic pathway. Indeed, resting NK cells largely express Fas ligand (FasL), while activated NK cells also express Tumor Necrosis Factor-related Apoptosis-Inducing Ligand (TRAIL), which engage death receptors expressed on the surface of target cells (43, 44). In melanoma cells, NK cell-mediated activation of the apoptotic extrinsic pathway is induced by the engagement of Fas (45, 46) (**Figure 1**).

Activated NK cells also release cytokines, particularly Interferon (IFN)γ and Tumor Necrosis Factor (TNF)α, exerting both direct and indirect effects contributing to target cells clearance (19). IFNγ is able to directly inhibit cell cycle progression and to promote apoptosis in infected and/or transformed cells as well as to increase their immunogenicity by enhancing antigen presentation (47). IFNγ also dampens proliferation and survival of endothelial cells, thus counteracting angiogenesis (48). Moreover, IFNγ broadly affects the immune system by stimulating the recruitment, differentiation and activation of several immune cell subsets involved in anti-viral and anti-tumoral responses (47). Similar effects have been observed for TNFα, albeit the activities of the two cytokines are not completely overlapping. Indeed, TNFα is considered to be poorly cytotoxic/cytostatic against tumor cells, while it is particularly effective in inducing inflammation and vasculature destruction (49).

The proper activation of NK cells is also supported by several cytokines produced by different immune cell subsets. Interleukin (IL)-2, IL-15, IL-12 and IL-18 as well as type I IFNs are known to promote several aspects of NK cell functioning, including proliferation, maturation, survival, cytokine secretion and up-regulation of activating receptors and cytotoxic molecules (50). Moreover, IFNγ can act as autocrine factor to stimulate NK cell cytotoxicity by up-regulating the expression of perforin, granzymes and FasL. Of notice, while most of the processes can be induced by individual cytokines, they represent a weak stimulus for IFNγ secretion, which instead requires the cooperation between IL-12 and the other cytokines (50).

## 3 Natural Killer Cells Within Melanoma TME

**Figure 2:**
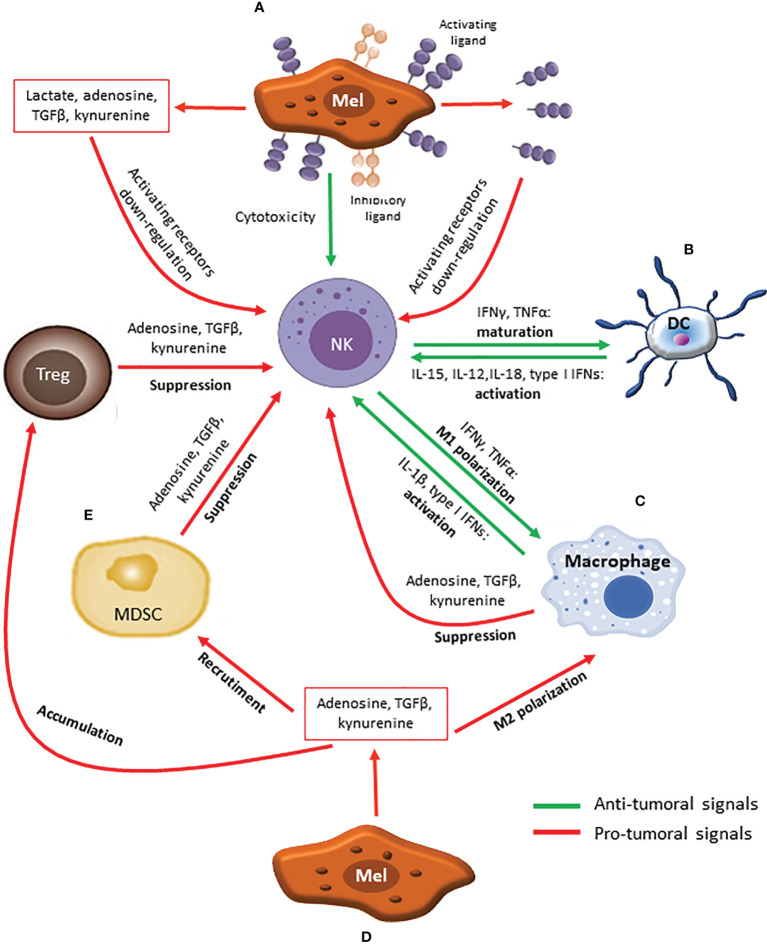
The complex cross-talk affecting the activity of NK cells within melanoma TME. **(A)** Melanoma cells phenotype, characterized by low levels of MHC class I molecules and high levels of activating ligands, promotes NK cells activation and killing. Additionally, melanoma cells can exploit both intrinsic (down-regulation/shedding of activating ligands) and extrinsic (secretion of immune suppressive molecules) mechanisms to escape from NK cell-mediated elimination. **(B)** Dendritic Cells (DCs) secrete cytokines such as IL-15, IL-12, IL-18 and type I IFNs which induce NK cells activation. In turn, activated NK cells produce IFNγ and TNFα, promoting DCs maturation. **(C)** Similar to DCs, M1 macrophages promote NK cells activation through the release IL-1β and type I IFNs, while IFNγ and TNFα secreted by activated NK cells further support M1 polarization. **(D)** Aside from direct effects against NK cells, immune suppressive molecules secreted by melanoma cells also induce M2 polarization, MDSCs recruitment and Tregs accumulation. **(E)** In turn, MDSCs and Tregs exploit suppressive molecules (Adenosine, TGFβ, kynurenine) to counteract NK cells activation.

Solid tumors consist of cancer cells and the surrounding tissue cells that constitute TME, which include several types of cells such as fibroblasts, endothelial and immune cells as well as extracellular matrix (ECM). In melanoma, the elevated mutational burden makes the tumor highly immunogenic. In addition, oncogenes commonly mutated in melanoma induce the expression of cytokines, chemokines, enzymes and growth factors recruiting and regulating immune cells (51). As a consequence, melanoma lesions usually show a substantial immune infiltrate composed by several distinct populations exerting specific, and even opposed, functions towards cancer cells. Moreover, the same subset can display different activities based on its state of maturation/polarization (52). The dynamic interactions between tumor and immune cells largely contribute to determine tumor progression. This complex interplay can be described through the immunoediting paradigm, that postulates an elimination phase in which the immune system efficiently eliminates cancer cells, an equilibrium phase in which immune response acts as an evolutive pressure selecting resistant clones, and an escape phase in which the tumor overcomes the immune response and successfully progresses (53). The immune escaping of melanoma cells can occur through two not mutually exclusive mechanisms: intrinsic phenomena reducing the capability of tumor cells to be recognized and killed by immune cells and extrinsic processes involving the active production of molecules generating, directly or indirectly, an immunosuppressive TME (54). Moreover, the capability of the TME to support or limit melanoma progression is affected by the relative proportion and contribution of the different immune cell subsets (52) (**Figure 2**). Many of these processes can be, at least partially, counteracted by standard chemotherapeutics, which provides the rationale for their combination with ICIs.

### 3.1 Chemotherapy Improves Melanoma Cells Recognition by NK Cells Within TME

Among the intrinsic mechanisms, both down-modulation and up-regulation of activating NK cell ligands have been reported. A reduced surface expression of activating ligands has been observed during melanoma progression, with metastatic lesions showing lower levels of these molecules compared to the primary counterpart (37, 38). Commonly, this lower expression has been attributed to ligands shedding, that is the generation of a soluble form of the molecule and its release in the extracellular space. The best-described ligands shed by melanoma cells are the NKG2D ligands MICs and ULBPs, but also the shedding of B7-H6, engaged by NKp30, has been reported. As expected, shed ligands show an opposite pattern compared to the surface counterpart, increasing in the course of disease (55–57). Although paradoxical, the over-expression of activating ligands has also been reported as an important mechanism to down-regulate NK cell cytotoxicity (58, 59). Both ligands shedding and over-expression culminate in the hyper-stimulation of the receptor which is in turn internalized to prevent excessive activation, thus resulting in NK cell desensitization and lower cytotoxic potential (55) (**Figure 2A**). Indeed, NK cells from melanoma patients often show a reduced expression of activating receptors and an impaired capability to respond to cancer cells (24, 60, 61).

DNA damage occurring in early tumorigenesis is recognized by the damage sensor kinases ATR and ATM, which in turn induce a kinase cascade involving downstream mediators such as the checkpoint kinases CHK1 and CHK2 and the tumor suppressor p53. The pathway is known as DNA damage response and is able to induce apoptosis and/or senescence, thus blocking cancer cell proliferation (62). This stress response also affects tumor cells susceptibility to NK cell-mediated elimination by inducing the expression of NKG2D ligands (63). The cytotoxic effects of DTIC and TMZ as well as cisplatin against melanoma cells rely on their capability to generate adducts within DNA and to induce the stress response, thus exerting an analogue immunomodulatory effect (62–64).

**Figure 3:**
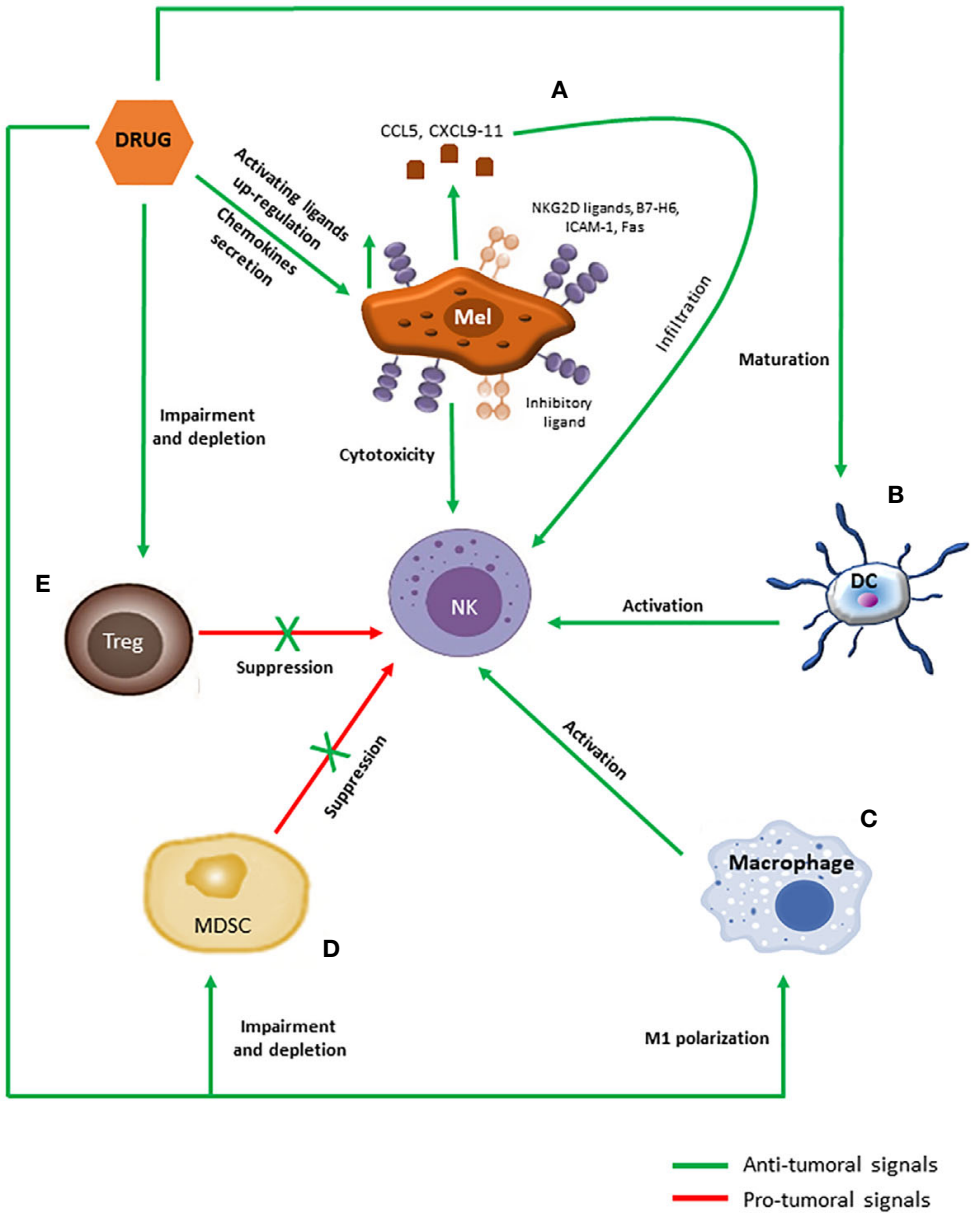
Immunomodulatory effects of anti-melanoma chemotherapeutics promoting NK cells activation. **(A)** Chemotherapeutic drugs induce the secretion of CCL5 and CXCL9-11 chemokines by melanoma cells as well as the up-regulation of activating ligands on their surface, which in turn promote NK cells infiltration and activation, respectively. **(B–E)** Moreover, chemotherapeutic drugs promote maturation in DCs **(B)** and M1 polarization in macrophages **(C)**, which support NK cells functioning through cytokines secretion and cell-to-cell contacts, and blunt accumulation and functions of MDSCs **(D)** and Tregs **(E)**, counteracting their inhibitory effects against NK cells.

The evidence that DTIC is able to up-regulate NKG2D ligands expression was observed in a melanoma murine model (**Figure 3A**), together with the needing of a competent immune system for the drug to exert its antitumor activity (65). Indeed, NKG2D ligands expression induced by DTIC enhanced both perforin/granzyme B-dependent killing of melanoma cells and IFNγ secretion by NK cells. In turn, IFNγ was mandatory to up-regulate MHC-I molecules expression and endogenous antigen presentation on melanoma cells, which allowed their recognition and elimination by cytotoxic T cells possibly through both perforin/granzyme- and Fas-mediated pathways (65, 66). A similar mechanism of action has been later reported also for TMZ (67).

Thus, NK cell targeting of melanoma cells sensitized by alkylating agents would be pivotal to trigger both innate and adaptive cytotoxic anticancer responses within TME, resulting in an effective restrain of tumor growth. Supporting this notion, NK cells expressing NKp46 have been found to be increased in DTIC-treated melanoma patients (68). Furthermore, DTIC responding patients displayed higher NK cell activation and cytolytic activity against melanoma cells (69).

A similar capability to increase NKG2D ligands expression has been described also for cisplatin, by which the drug can enhance NK cells cytotoxicity. In addition, cisplatin-induced up-regulation of other activating molecules such as B7-H6, ICAM-1 and Fas has been reported (70–75) (**Figure 3A**). Moreover, cisplatin can sensitize tumor cells to granzyme B by augmenting tumor cell permeability and by increasing the expression of the granzyme-target caspase-3, which mediates the execution phase of apoptosis (76, 77).

Effective killing relies on a functioning cytoskeleton in order to mediate the correct aggregation of signaling receptors and adhesion molecules as well as the polarized movement of cytotoxic granules at the immune synapse (78). Since PTX interferes with microtubules dynamics, it is expected to dampen NK cell cytotoxicity, as demonstrated by several authors (79–81). However, other reports challenged these observations, indicating that PTX might actually enhance NK cell-mediated elimination of tumor cells by increasing NK cell cytotoxicity, by inducing the expression of ICAM-1 and MIC-B and/or by sensitizing melanoma cells to killing (76, 81–84) (**Figure 3A**). These apparently contradictory results probably depend on the amounts of PTX used in the different experimental settings, since immunomodulation is usually induced by low doses of chemotherapeutics, while high doses are considered to be frankly immunosuppressive (85).

The immunological effects induced by DTX are largely overlapping with those observed for PTX. DTX effects on NK cell-mediated killing are variable, with some authors reporting a suppression while others showed that DTX enhanced NK cell cytotoxicity (81, 86). The underlying molecular mechanisms appeared to be shared with PTX, involving an increased expression of NKG2D ligands and ICAM-1 (86, 87) (**Figure 3A**). However, the DTX-mediated up-regulation of Fas has also been reported, suggesting that the two taxanes may also elicit different pathways (88).

Collectively, these evidences indicate that drugs used in melanoma chemotherapy possess the capability to up-regulate the expression of NK cell activating ligands on melanoma cells, thus improving their NK-cell mediated recognition and killing, an immunomodulatory activity that may contribute, together with direct cytotoxicity, to the anti-tumoral effects of chemotherapeutics. Moreover, while the main targets of such up-regulation appear to be NKG2D ligands, different drugs have been also shown to induce different additional activating ligands such as B7-H6, ICAM-1 and Fas. These non-overlapping activities could be effectively used to develop patient-tailored therapies in which the choice of the chemotherapeutic to be used is driven by the specific melanoma immune phenotype.

### 3.2 Chemotherapy Facilitates NK Cells Infiltration Within TME

In order to effectively eliminate targets, killer cells must first of all make contact with them. As previously mentioned, NK cells are effective in eliminating circulating melanoma cells (20, 24, 25) but their activity against the tumor mass is strongly limited by their poor capability to infiltrate melanoma TME (22, 23). Thus, strategies aimed to improve NK cells infiltration would represent valuable approaches to exploit their natural cytotoxicity.

In addition to directly increase the susceptibility to immune-mediated clearance of melanoma cells, alkylating drugs have been shown to also affect immune cells infiltration within TME. In a mouse model of spontaneous melanoma, both DTIC and TMZ have been shown to induce the secretion of chemokines such as CCL5, CXCL9, CXCL10 and CXCL11 by melanoma cells (**Figure 3A**). Effector T cells expressed the cognate receptors CCR5 and CXCR3, thus the augmented chemokines release promoted robust infiltration of anti-tumor T cells within melanoma TME, which correlated with a better disease control and survival also in melanoma patients (89, 90). NK cells are equipped with the same receptors as effector T cells, thus they might be recruited within melanoma TME through the same pathway (21). Similar findings have been reported for cisplatin and DTX, which promoted lymphocyte recruitment and infiltration within tumor by inducing melanoma cells to express CXCL10 and CXCL11, respectively (91, 92) (**Figure 3A**).

On the other hand, the mechanism by which PTX recruits effector cells has been shown to be different. Indeed, PTX promoted lymphocytes migration and infiltration within melanoma TME by down-regulating CD62L, a lymph node homing receptor also expressed by NK cells (93, 94).

Another factor affecting lymphocyte infiltration is the composition of tumor stroma, particularly the density of collagen fibers which limit chemokines availability (90). In this context, DTIC-sensitive melanoma lesions have been shown to up-regulate genes involved in ECM remodeling, suggesting that the drug may trigger two different pathways synergizing to promote the effective recruitment of cytolytic cells (95).

Overall, the changes induced by chemotherapy in stroma composition may facilitate the migration of NK and other killer cells within TME, helping to overcome one of the main problem of ICIs treatment, that is the poor infiltration of effector cells. These evidences also suggest the assessment of NKG2D ligands, chemokines and/or ECM components within TME as possible strategies to predict and/or follow the therapy outcome.

### 3.3 Chemotherapy Selectively Targets Immune Suppressive Cells Affecting NK Cell Activity Within TME

As previously mentioned, immune infiltrate within tumors is highly heterogeneous, being composed by different subpopulations with distinct activities. Immune system components are profoundly interconnected each other, thus several subsets can interact with NK cells (**Figure 2**). In addition, their activity can be affected by tumor cells. The net result of this complex connections plays a pivotal role in determining tumor progression (96), thus therapeutic approaches able to shift the balance toward anti-tumor activities would contribute to melanoma elimination by the immune system. In this context, standard chemotherapeutics may represent a valuable tool due to their capability to preferentially target immune cell subsets inhibiting NK cells and supporting the generation of a suppressive TME.

#### 3.3.1 Dendritic Cells

Dendritic Cells (DCs) are tissue-resident innate immune cells characterized by a marked variability in terms of phenotype and functional properties. While mature DCs (mDCs) play a positive role against tumors thanks to their capability to induce adaptive responses and to secrete immune-stimulating cytokines, immature DCs (iDCs) instead display tolerogenic properties, promoting tumor escape (97). TME features such as hypoxia and acidosis are common to inflamed tissues and thus promote DCs maturation and anti-tumor responses thanks to the secretion of IFNγ and IL-12 (98, 99). However, other molecules produced by tumor cells such as lactic acid, suppressive cytokines and adenosine prevent DCs recruitment and differentiation (100–103). In turn, iDCs express TGFβ, IL-10 and IDO, thus generating a positive loop that maintains the immunosuppressive milieu (100–103).

DCs represent one of the major immune subsets recruiting and triggering NK cells. By expressing the same range of chemokines secreted by melanoma cells, DCs are able to attract NK cells within TME (104). Moreover, DCs produce several cytokines involved, together with cell-to-cell contact, in NK cell activation. Particularly, DCs express IL-15 promoting NK cells proliferation, survival and activation; IL-12 and IL-18 needed to stimulate cytokines secretion as well as cytotoxicity; and type I IFNs which stimulates killing (105). In turn, cytokines secreted by NK cells, such as IFNγ and TNFα as well as NKp30 engagement, support DCs maturation and polarization towards an anti-tumor phenotype (106, 107) (**Figure 2B**). In addition, NKp30, together with NKp46 and DNAM-1, mediates DCs editing, allowing recognition and killing of iDCs (108, 109). The choice between induction of maturation or killing appears to be dependent on NK/DC ratio: if NK cells are preponderant, they tend to kill iDCs, while maturation is induced when DCs prevail (106, 107). Additionally, mDCs are spared thanks to the high levels of MHC-I molecules that act as inhibitory signals (108, 109). In this way, NK cells select DCs able to properly present antigens. Antigen presentation by DCs can be further supported by NK cells killing, which may induce the release of antigens and/or danger signals (110). However, iDCs editing can be prevented by TGFβ secreted by both iDCs and melanoma cells, which determines NKp30 down-regulation (111).

Taxanes and cisplatin have been shown to promote DCs motility, maturation and activation through the stimulation of toll-like receptor (TLR) 4, a typical pattern recognition receptor (PRR) expressed by myeloid cells (112–116) (**Figure 3B**). However, although similar in the engaged pathway as well as the general effects exerted on DCs, the activation mediated by the two classes of drugs relies on different mechanisms and ends up in the secretion of different cytokines. Taxanes directly stimulate TLR4 by mimicking its natural ligand lipopolysaccharide and mainly induce the secretion of IL-12 (112, 113, 117). On the other hand, cisplatin-induced maturation of DCs has been proposed to be indirect, due to the capability of this drug to trigger immunogenic cell death, a peculiar type of apoptosis in which cell death is accompanied by the release of endogenous danger signals (115, 118). As a consequence, DCs are induced to mature by such danger molecules mainly secrete type I IFNs (115, 116).

Since DCs are the main immune subset involved in early NK cells recruitment and activation, their chemotherapeutics-induced maturation would represent an effective mechanism to boost NK cells functioning within TME. In turn, the cross-talk between activated NK cells and mDCs would be pivotal to generate more effective adaptive anti-tumoral responses.

#### 3.3.2 Macrophages

Tumor Associated Macrophages (TAMs) are the most abundant leukocyte subpopulation infiltrating tumors. Similar to DCs, macrophages are characterized by high plasticity in response to environmental stimuli and can display a huge range of phenotypes and functions whose extremes are represented by two opposed states of polarization. Classical M1 macrophages are pro-inflammatory cells able to present antigens and to secrete anti-tumor Th1 cytokines, while alternatively-activated M2 macrophages promote immune suppression, angiogenesis and tissue remodeling (119). In melanoma, TAMs mainly derive from circulating monocytes recruited by hypoxia as well as by pro-inflammatory cytokines secreted by melanoma cells, DCs and tissue-resident macrophages (120).

NK cells cross-talk with macrophages largely recapitulates phenomena occurring with DCs. Similar to mDCs, M1 macrophages promote NK cell activation and cytolytic activity against tumor cells and iDCs by releasing IL-1β and type I IFNs, which up-regulate NCRs and NKG2D, respectively. In addition, the physical interaction between NK cells and macrophages, mainly mediated by DNAM-1, induces IFNγ release which in turn promotes M1 polarization (121–123) (**Figure 2C**). Of notice, type I IFNs also stimulate IL-15 cis-presentation on NK cells, that further induces IFNγ secretion (121). Moreover, activated NK cells are able to edit macrophage pool by selectively killing M2 subset through the engagement of NKp46 and DNAM-1. Again, M1 macrophages appear to be cytotoxicity-resistant thanks to the high expression of MHC-I molecules (123, 124). In melanoma models, monocytes and M1 macrophages have been shown to play an important role in recruiting and priming NK cells in the TME in order to prevent metastasization (125, 126). However, the hypoxic and acid TME largely contributes to monocytes switching towards an M2 phenotype, a phenomenon that is further supported by molecules expressed by either melanoma and stromal cells such as adenosine, IL-10 and TGFβ (127–130). In turn, M2 macrophages produce IL-10, TGFβ and IDO, which further support M2 polarization, dampen DCs maturation and widely suppress NK cell functions (120) (**Figure 2D**). Indeed, TAMs are usually associated with a poorer outcome in melanoma (131).

Similar to what has been described for DCs, taxanes affect macrophages polarization and function by engaging TLR4 (117). In murine macrophages, PTX-mediated stimulation of TLR4 not only induced the acquisition of an M1 phenotype, as indicated by the secretion of TNFα and IL-12 but was also able to counteract M2 polarization and to revert melanoma TAMs towards an M1 profile, which in turn caused melanoma regression (132, 133). DTIC has also been demonstrated to counteract M2 polarization and activity of melanoma TAMs by reducing PD-L1 expression and CCL22 secretion, although the underlying molecular mechanism is unknown (134) (**Figure 3C**).

On the other hand, cisplatin has been shown to induce the secretion by tumor cells of soluble factors that promoted macrophages polarization towards a M2 phenotype, which in turn may favor cancer cells migration (135, 136). Nevertheless, these effects could be counteracted by the higher sensitivity of M2 macrophages to cisplatin-induced apoptosis compared to M1 macrophages and DCs (136). Similar findings have been reported also for PTX (137, 138).

Compared to DCs, the effects of chemotherapy on macrophages polarization are less defined and partially conflictual, suggesting that the same drug could activate different pathways in different cell subsets. Still, the balance appears to lean in favor of M1 polarization, which would contribute to generate an anti-tumoral TME supporting the cytotoxic activities of both NK and T cells.

#### 3.3.3 Myeloid-Derived Suppressor Cells

While DCs and macrophages can establish both positive and negative interactions with melanoma and NK cells, Myeloid Derived Suppressor Cells (MDSCs), the third myeloid subpopulation composing TME, are outright pro-tumoral and suppressive. MDSCs are immature precursors of DCs, macrophages and neutrophils whose presence within melanoma TME is due to the chronic release of pro-inflammatory molecules by tumor cells, which recruit MDSCs from the bone marrow blocking at the same time their maturation and inducing their polarization towards suppressive cells (139) (**Figure 2D**). Thus, MDSCs generation represents a physiological mechanism aimed to prevent immune system over-activation and tissue damage that is subverted by melanoma cells in order to support tumor development. Indeed, MDSCs have been proven to enhance malignant properties of melanoma cells, including proliferation, epithelial-to-mesenchymal transition (EMT), dissemination and metastasization (140, 141). Moreover, MDSCs dampen anti-tumor functions of other myeloid cells by inhibiting DCs maturation and enhancing M2 switching (139). The suppressive activities of MDSCs rely on several mechanisms also exploited by melanoma cells, such as IDO up-regulation, PD-L1 expression, TGFβ secretion and adenosine production (142). The induction of such suppressive pathways by MDSCs is further enhanced by the hypoxic and acid conditions of TME (141, 143). Thus, MDSCs largely contribute to maintain the immune suppressive TME firstly generated by melanoma cells.

NK cells cross-talk with MDSCs ends up with the suppression of the former. MDSCs have been shown to inhibit NK cell cytolytic activity and IFNγ secretion through cell-to-cell contacts mostly mediated by membrane-bound TGFβ, which induce the down-regulation of NCRs and NKG2D expression both *in vitro* and in murine models (144–146) (**Figure 2E**). Moreover, IFNγ secreted by NK cells can activate a negative loop by enhancing TGFβ production by MDSCs (147). In addition, acid conditions found in TME can further increase MDSCs suppressive capabilities against NK cells (148) (**Figure 2D**).

The capability of taxanes and cisplatin to dampen the accumulation and suppressive properties of MDSCs has been well described in melanoma (116, 149–153) (**Figure 3D**). Moreover, PTX have been also shown to promote their maturation toward a DC phenotype (149, 150).

Since MDSCs immune suppressive activities largely affect the functions of the other immune cells, particularly myeloid cells, the capability of chemotherapeutic drugs to selectively deplete this subset may largely contribute to restore a Th1 milieu within TME and tumor elimination by cytotoxic effector cells.

#### 3.3.4 Regulatory T Cells

Regulatory T cells (Tregs) represent the most important suppressive immune cell subset within TME. Tregs are adaptive lymphocytes able to counteract the activation of all the immune cells in order to prevent autoimmunity and maintain self-tolerance. However, they are also widely recruited within TME by chemokines and hypoxia in order to promote immune evasion (**Figure 2D**). In addition, Tregs accumulation can also rely on the local expansion of infiltrating Tregs as well as on the trans-differentiation of conventional T cells, both mediated by IL-10 and TGFβ (154). Indeed, Tregs presence within melanoma TME has been associated with tumor progression and poor survival (155). Once activated, Tregs can directly eliminate killer cells through the perforin/granzyme-mediated pathway, suppress their activity by producing TGFβ, IL-10 and adenosine and/or limit their survival by depleting IL-2. Additionally, Tregs-released cytokines and cell-to-cell contacts largely contribute to dampen DCs maturation, promote MDSCs expansion and induce IDO expression on myeloid cells (156–158). In turn, iDCs and MDSCs can further stimulate Tregs functions through TGFβ (159, 160).

Data on the interactions between NK cells and Tregs in TME are limited, but they appear to overlap those observed for MDSCs. Degranulation, IFNγ release and expression of NKp44 and NKG2D by NK cells have been shown to be impaired by Tregs (**Figure 2E**). Again, such inhibition appears to be induced by the membrane-bound TGFβ expressed by Tregs (160, 161).

All the classes of drugs used in metastatic melanoma chemotherapy have been shown to preferentially target Tregs compared to the other lymphocytes (**Figure 3E**). However, the mechanisms employed to deplete Tregs are different and not completely overlapping. Alkylating agents have been demonstrated to affect Tregs by reducing their infiltration within TME. Particularly, DTIC acted by inhibiting PD-L1 expression and CCL22 secretion by M2 macrophages (134), while TMZ reduced the production of CCL2 by tumor cells (162).

Regard taxanes, several reports have shown that PTX treatment at low doses markedly impaired Tregs viability, suppressive functions and infiltration rates within TME, while cytotoxic lymphocytes were poorly affected (149, 163, 164) (**Figure 3E**). The molecular mechanism underlying this depletion appeared to be TLR4-indipendent and to involve instead an increased tendency to apoptosis due to the PTX-mediated up-regulation of Fas and/or alteration in the balance between pro- and anti-apoptotic factors in Tregs (165, 166). DTX activity against Tregs recruitment has also been widely reported, especially in tumor models but also in clinical settings (167–169) (**Figure 3B**). For this drug, the proposed mechanism of action resembled the one observed for alkylating agents, relying on its capability to dampen tumor cells secretion of CCL20, recognized by the CCR6 receptor expressed on Tregs (170). Cisplatin also appeared to be cytotoxic for Tregs since its usage is associated with reduced Tregs numbers in animal models, although the underlying molecular mechanism is not defined (171–173) (**Figure 3E**).

Considering the wide role of Tregs in inhibiting all the anti-tumoral components of the immune system stimulating at the same time those subsets equipped with suppressive properties, their selective elimination probably represents the most important immunomodulatory property of drugs used in metastatic melanoma chemotherapy.

## 4 The Use of Nanosystems to Improve the Effect of Chemotherapeutics in Melanoma

Nanomedicine represents an alternative strategy to deliver anti-neoplastic agents. Nanotechnology-based drug delivery systems act to improve effectiveness of chemotherapeutics in terms of bio-distribution, water solubility, targeting capability and therapeutic index. Many different types of nanosystems have emerged in the last years, empathizing their important role in the treatment of solid tumors, especially melanoma (174, 175). To facilitate their accumulation within TME, drugs can be encapsulated, adsorbed or covalently attached on nanocarriers. Furthermore, in the past decades, nanocarriers have been used to target TME in order to inhibit it suppressive capability by modulating immune cells, tumor stroma, cytokines and enzymes (176).

### 4.1 General Features of Nanocarriers

In recent years, several types of nanocarriers have been developed exploiting both organic and inorganic molecules (177–180) as well as natural vesicles (181, 182). Physical and chemical properties of nanocarriers affect their bio-distribution, internalization and degradation (183, 184). Additionally, they facilitate drug release and avoid accumulation and toxicity (185). Many strategies have been identified to prevent nanocarrier opsonization and clearance by Reticuloendothelial System (RES), thus increasing circulation time in blood stream (186). Among them, surface decoration with polyethylene glycol (PEG) or the generation of erythrocytes-membrane coated nanocarriers have been shown to be particularly effective (187, 188). Moreover, the PEG shell can be functionalized in order to target molecules specifically expressed by tumor cells or TME (189). Complement recognition and opsonization further depend on nanocarriers shape, which also plays an important role in their bio-distribution and circulation and ensures their penetration in the different skin layers (190).

**Figure 4:**
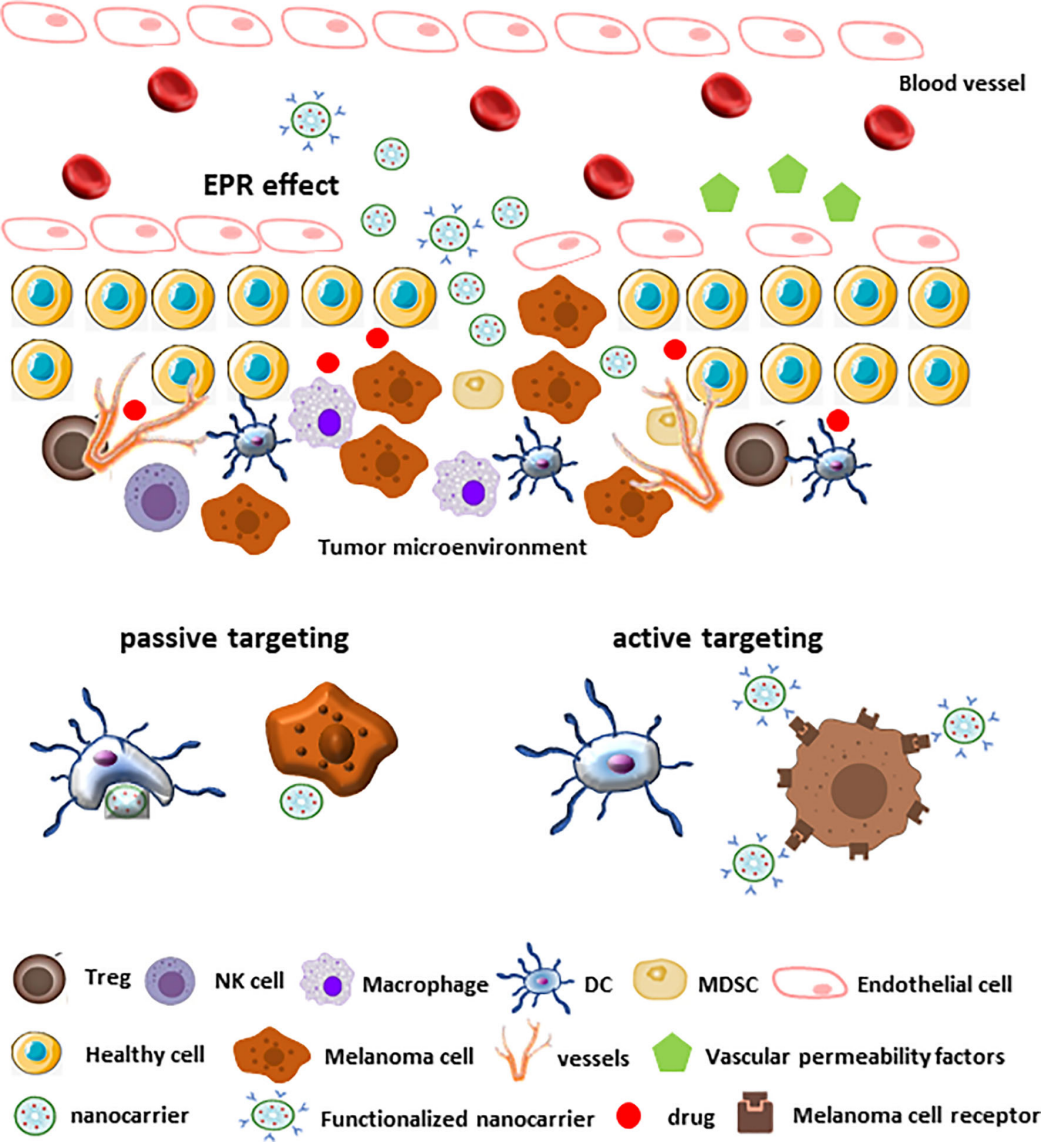
Passive and active targeting of nanocarriers within melanoma TME. Under certain conditions (e.g., hypoxia and inflammation) different factors (bradykinin, nitric oxide, prostaglandins, VEGF, cytokines) induce an increased permeability of the endothelium of tumor blood vessels. The rapidly growing tumor is facilitated by new vessels formation that contribute to the permeation of nanocarriers to the tumor stroma. Furthermore, the absence of normal lymphatic drainage in tumors contribute to the nanocarriers retention (EPR effect). In these conditions, the passive uptake of nanocarriers is improved (passive targeting). On the contrary, the decoration of the nanocarrier surfaces with ligands that recognize the tumor cell receptors facilitates the drugs delivery in melanoma TME.

Specific targeting of tumor TME by nanocarriers can be achieved by two non-mutual strategies: passive and active targeting. Passive targeting relies on the Enhanced Permeability and Retention (EPR) effect, which in turn depends on the typical features of tumor neo-vascularization (**Figure 4**). Tumor angiogenesis leads to high vascular density aimed to ensure a sufficient amount of nutrients and oxygen to tumor tissues. Furthermore, the large gaps between endothelial cells in tumor blood vessels facilitate the extravasation. Together, the two mechanisms allow the penetration of nanocarriers within TME (191, 192) (**Figure 4**). At the same time, tumors are unable to develop lymphatic vessels *de novo*, which results in poor drainage promoting nanocarriers retention (193). Furthermore, most solid tumors, including melanoma, show high levels of vascular permeability mediators that can further enhance EPR effect and drug delivery (192) (**Figure 4**).

On the other side, active targeting aims to facilitate the interaction between drug-loaded nanocarriers and target cells, reducing non-specific drug interactions. This mechanism depends on nanocarriers surface functionalization with ligands, such as mAbs or peptides, able to recognize with high affinity receptors and/or molecules expressed on specific target cells, thus conferring more specificity to the delivery system (178) (**Figure 4**).

In this context, NK cells have been proved to be a valuable source of targeting molecules for tumor cells. Indeed, NK cell activating receptors can be fused to drug-loaded nanocarriers to form NKsomes, which are able to target cancer cells thanks to the high expression of activating ligands (194).

### 4.2 Use of Nanocarriers to Deliver Chemotherapeutics and Target Immune Cells Within Melanoma TME

TME takes part in cancer development, proliferation and metastasis as well as immune escape and contributes to the failure of many conventional cancer therapies (53, 54). Nanomedicine has been proposed as a new therapeutic strategy to modulate TME components to increase cancer chemotherapy performance (195, 196).

Because of EPR effect, nanocarriers accumulate inside TME after intravenous administration and are retained for long periods (197). Aside from tumor cells, nanocarriers can be acquired also by the other cells constituting TME, particularly by tumor-infiltrating phagocytes. Among them, DCs are the cell type equipped with the widest range of uptake mechanisms (198). Thus, they represent the best targetable immune cell subset within TME. Particularly, nanocarriers with a size between 20 to 200 nm enter in the capillaries, are retained in the draining lymph nodes and are taken up by resident DCs, while those with a size range of 500-2000 nm are taken up by local DCs at the site of injection. Therefore, smaller nanocarriers size correlates with higher DC uptake. Additionally, positively charged nanocarriers are more actively taken up by DCs, most probably due to the negative charge of ECM that immobilizes nanocarriers. On the contrary, negatively charged nanocarriers may be cleared by RES or opsonized by the complement system (199).

From a pharmacological point of view, chemotherapeutics used in metastatic melanoma circulate into the body through blood stream and penetrate into the tumor by passive diffusion. This causes a weak delivery of the drug with non-specific distribution, low response, no overall survival benefit and drug toxicity (1).

In recent years, the application of nanotechnologies became an efficient strategy to improve the effectiveness of chemotherapeutic agents for melanoma treatment (200). Particularly, nanocarriers appeared to be more advantageous in comparison with traditional chemotherapeutics for their capability to encapsulate lipophilic drugs, to provide higher stability and longer circulation time in the bloodstream as well as to control drug release (189). These innovative strategies increased drug accumulation into TME (EPR effect) compared with the poor solubility, penetration capability and bio-availability of standard chemotherapeutic drugs. Moreover, the decoration of nanocarrier shells with targeted ligands (active targeting) facilitates the interaction with tumor cells (**Figure 4**).

Several nanosystems for the delivery of therapeutics at melanoma site have been studied. They include lipid and polymer-based nanoparticles designed to improve delivery and release of drugs to melanoma cells, thus exhibiting robust effects against melanoma cells proliferation and angiogenesis both *in vitro* and *in vivo* (201, 202). In addition, nanocarriers, if combined with photothermal/photodynamic therapy, specifically target melanoma cells, accumulate within melanoma TME and inhibit tumor growth more efficiently than the free chemotherapeutic agents (203, 204). To overcome PTX poor water solubility, new drug delivery strategies have been investigated (205). Notably, liposomal systems able to directly deliver PTX to melanoma cells have been developed and studied both *in vitro* and *in vivo*. Liposomes allow a sustained release of PTX that is not affected by low pH, a condition commonly observed in melanoma TME. Furthermore, liposomes surface can be functionalized by adding peptides able to both recognize integrins expressed by melanoma cells and promote low pH-mediated internalization of liposomes, with increased anti-tumoral effects and survival compared to free PTX (206). Moreover, liposomes can be functionalized with Fibroblast Growth Factor (FGF)-derived peptides to improve melanoma cells targeting and favor their accumulation within TME (207).

One of the most important barriers which compromises the efficacy of nanocarriers is associated with the ability of immune cells to recognize and engulf nanoparticles. To overcome this problem, the possibility to deliver immunoregulating agents for specific immune cell populations has been investigated (208). This combination is useful to activate killer cells facilitating the elimination of metastatic cells during the invasion and metastasization process and could potentiate the efficacy of immunotherapy by increasing delivery and retention and by reducing immunomodulation toxicity (196, 209). Nanocarriers for the delivery of IL-12 are generated to improve tumor infiltration of killer cells, while other peptides may specifically modulate T cells proliferation, infiltration, and activation (209). Furthermore, PLA microspheres carrying IL-12 or TNFα have been developed to overcome the suppressive effects of the TME. In particular, the injection of microspheres in B16 melanoma-bearing mice provoked the induction of a tumor-specific memory T cell response and consequently the tumor rejection (210).

Recently, the role of NK cell-derived exosomes has gained attention since they are equipped with the same cytotoxic molecules found in NK cells, such as perforin and FasL. Therefore, they can eliminate tumor cells by the same mechanisms as NK cells. In melanoma, NK cell-derived exosomes have been shown to induce apoptosis *in vitro* as well as to limit tumor growth *in vivo* (211). Taken together, these data underlie the role of nano-based drug delivery systems combined with chemoimmunotherapy as a future strategy to treat metastatic melanoma by boosting immune response.

## 5 Discussion

In the clinical practice, the introduction of ICIs reactivating T cell-mediated immune responses against metastatic melanoma has dramatically changed patients’ outcome. However, the failure of a fraction of patients to respond to therapy spurred the investigation of novel approaches in order to improve the success rate. In this context, NK cells gained increasing attention because of their alternative and complementary capability to kill tumor cells compared to T cells. Most of the ICIs that are currently used or under development target inhibitory receptors also expressed by NK cells. Indeed, it has been shown that their blockade is able to reactivate NK cell-mediated cytotoxicity and that NK cells are essential for ICIs anti-tumoral activity (212). Based on these evidences, ICIs targeting NK cell inhibitory receptors such as NKG2A and KIRs have been developed and tested in clinical trials (213). An alternative strategy to improve NK cell-mediated killing is the usage of bi- and trispecific Killer Cell Engagers, which engage activating receptors on NK cells and specific antigens on tumor cells, thus favoring immune synapse formation and NK cell degranulation (212).

In addition, NK cells have been evaluated for adoptive cell transfer both in autologous and allogenic settings. Of notice, NK cells to transfer can be modified to express a Chimeric Antigen Receptor (CAR). Initially developed for T cells to allow them to recognize naïve antigens, CAR technology can be applied also to NK cells to redirect them against specific targets. Compared to CAR-T cells, CAR-NK cells are not antigen-restricted and maintain their capability to recognize the target through multiple pathways, ensuring effective activation. Moreover, CAR-NK cells are short-living cells that do not generate memory, thus displaying a very low risk of adverse reactions and a high safety profile (214). However, when used in solid tumors, these approaches suffer from the same limits observed for T cells, that is the presence of an immune suppressive TME that limits NK cell trafficking within the tumor and/or blunts their cytotoxic activities.

The usage of chemotherapeutics to treat cancer relied on their capability to counteract uncontrolled tumor cells proliferation. However, their action is not cell-specific, which means that all the cell types with high rates of replication, including immune cells, are negatively affected by chemotherapy. Indeed, immune suppression is one of the most important collateral effect of standard-dosage chemotherapy and thus was regarded as the major mechanism making chemotherapy incompatible with immunotherapy. However, more recent evidence challenged this assumption, showing that sub-toxic concentrations of chemotherapeutics exert immunomodulatory activities able to reactivate immune responses. Additionally, it has been shown that suppressive immune subsets display a higher sensitivity to chemotherapy-mediated cytotoxicity, making low doses of chemotherapeutics a potential strategy to deplete specific immune cell populations (85). Thus, the usage of chemotherapy is nowadays gaining attention as therapeutic strategy to improve effectiveness rates of current immunotherapy.

The evidence discussed here indicate that metastatic melanoma chemotherapy affects not only tumor cells but also the TME, thus having the potential to unleash the anti-tumor activities of the immune system. However, the combination between chemo- and immunotherapy has recently begun to be investigated and there is still not a consensus about the schedule to be used. Indeed, while some reports suggested that chemotherapy should be administrated before immunotherapy (11–13), other studies demonstrated that the survival benefit could be achieved also by the concomitant administration of the two kinds of drugs (14, 15). Moreover, some case reports indicated that chemotherapy could be effectively used after immunotherapy in non-responding patients (16, 17). Although in theory initial chemotherapy would represent the best strategy to reactivate the immune system and sensitize patients to immunotherapy, the lack of biomarkers predicting immunotherapy response makes the subsequent usage of chemotherapy a more feasible approach. Overall, additional studies are needed in order to define the best therapeutic strategy to combine chemo- and immunotherapy as well as to identify those patients actually needing the combinatorial therapy.

NK cells within melanoma TME can directly kill tumor cells and/or promote anti-tumoral activities by other immune subpopulations. However, cell-intrinsic pathways in melanoma cells as well as their capability to recruit and activate suppressive subsets can profoundly impair NK cells cytolytic and anti-proliferative functions. The evidences reported here clearly suggest that chemotherapeutic drugs may support NK cell-mediated elimination of melanoma cells by four different, complementary mechanisms: 1) increase of melanoma cells immunogenicity, mainly occurring through the up-regulation of NKG2D ligands but also involving other activating molecules specifically induced by the different drugs, thus boosting the natural cytotoxic activity of NK cells; 2) induction of NK cells infiltration within TME, which would contribute to overcome an important limitation in the usage of autologous, allogenic as well as CAR-NK cells; 3) promotion of myeloid cells activation and differentiation towards anti-cancer phenotypes that largely contribute to develop and support NK cells functions; 4) specific impairment and depletion of immune suppressive cells, which play the major role in generating and maintaining the suppressive conditions blunting NK cells cytotoxicity within TME.

Moreover, these effects could be further enhanced by nano-based drug delivery systems designed to improve pharmacokinetics behaviors and to increase drugs stability *in vivo*, thus allowing the effective usage of sub-toxic amounts of drugs that keep the immunomodulatory properties avoiding at the same time the immune suppression commonly associated to high-dosage of chemotherapeutics (215).

Overall, the association of immunotherapies with low dosages of chemotherapeutics possibly delivered by nanosystems could restore NK cells anti-tumor properties thus representing an effective alternative therapeutic strategy for those melanoma patients that fail to respond to the current immunotherapeutic treatments.

## Author Contributions

CMC conceived the work. CG and CMC reviewed the relevant literature. CG, CDM and CMC wrote the paper. CG, CDM, and CMC generated the figures. All the authors critically read and revised the manuscript, contributed to the article and approved the submitted version.

## Funding

This work was supported by Ministero dell’Istruzione, dell’Università e della Ricerca (MIUR) PRIN 2017 2017M8YMR8_002.

## Conflict of Interest

The authors declare that the research was conducted in the absence of any commercial or financial relationships that could be construed as a potential conflict of interest.

## Publisher’s Note

All claims expressed in this article are solely those of the authors and do not necessarily represent those of their affiliated organizations, or those of the publisher, the editors and the reviewers. Any product that may be evaluated in this article, or claim that may be made by its manufacturer, is not guaranteed or endorsed by the publisher.
